# Model of succession in degraded areas based on carabid beetles (Coleoptera, Carabidae)

**DOI:** 10.3897/zookeys.100.1534

**Published:** 2011-05-20

**Authors:** Axel Schwerk, Jan Szyszko

**Affiliations:** Laboratory of Evaluation and Assessment of Natural Resources, Warsaw University of Life Sciences, Nowoursynowska Street 166, 02-787 Warsaw, Poland

**Keywords:** Carabidae, degraded areas, logistic equation, Mean Individual Biomass (MIB), succession

## Abstract

Degraded areas constitute challenging tasks with respect to sustainable management of natural resources. Maintaining or even establishing certain successional stages seems to be particularly important. This paper presents a model of the succession in five different types of degraded areas in Poland based on changes in the carabid fauna. Mean Individual Biomass of Carabidae (MIB) was used as a numerical measure for the stage of succession. The run of succession differed clearly among the different types of degraded areas. Initial conditions (origin of soil and origin of vegetation) and landscape related aspects seem to be important with respect to these differences. As characteristic phases, a ‘delay phase’, an ‘increase phase’ and a ‘stagnation phase’ were identified. In general, the runs of succession could be described by four different parameters: (1) ‘Initial degradation level’, (2) ‘delay’, (3) ‘increase rate’ and (4) ‘recovery level’. Applying the analytic solution of the logistic equation, characteristic values for the parameters were identified for each of the five area types. The model is of practical use, because it provides a possibility to compare the values of the parameters elaborated in different areas, to give hints for intervention and to provide prognoses about future succession in the areas. Furthermore, it is possible to transfer the model to other indicators of succession.

## Introduction

Nowadays, there is a rising awareness of our natural resources. Management of natural resources seems to be a key element of sustainable development. Among the many types of ecosystems and habitats, degraded areas have acquired special importance, particularly with respect to restoration measurements. A crucial aspect of many degraded areas seems to be a serious loss in biodiversity (Dobson et al. 1997). Consequently, the improvement of habitat conditions in degraded areas is often required to restore or maintain animal and plant communities. Careful management of successional stages is important with respect to the future development and faunal and floral post-disturbance recovery (e.g. Bradshaw 1984; Tilman 1987; Jochimsen 2001). This may imply the facilitation or inhibition of the successional process (e.g. Bradshaw 1983, 1984; Dobson et al. 1997). To carry out restoration efforts in an ecologically sound way, knowledge about processes of succession in degraded areas is obviously necessary.

Numerous publications focus on the mechanisms and phases of successional processes. Alternative pathways of succession are described in the models of Connell and Slatyer (1977) and Abrams et al. (1985). Drechsler et al. (2009) describe a disturbance-succession model combined with a reserve selection algorithm as a tool for conservation planning. Complex mathematical models of succession are DRYADES (Mailly et al. 2000) and LANDIS (He et al. 2005; Scheller et al. 2007). However, for practical applications often more simple models are needed, which require relatively little effort in terms of data input.

The aim of this paper is to present a simple model established by Schwerk (2008), which allows for a description and comparison of the successional process on different types of degraded areas. The model will be constructed in two steps. Firstly, based on comprehensive field studies the basic parameters of succession in degraded areas will be extracted and a conceptual model will be described. Since carabid beetles are sensitive to alterations of environmental conditions (Rainio and Niemelä 2003), changes in carabid beetle fauna will be used as indicator of the succession process. In a second step these parameters will be specified mathematically. Thus, it will be possible to quantitatively express differences in succession between different types of degraded areas.

## Methods

### Elaboration of field data

Field data were collected at five different types of degraded areas in Poland, namely planted stands on forest soil, naturally regenerated stands on post-agricultural soil, planted stands on post-agricultural soil, planted stands on a heap of ashes produced by a power station and planted stands on a brown coal mining heap. All of these areas may be regarded as being degraded due to intense forestry or agriculture in the past or due to their anthropogenic origin. This results in decreasing timber, ineffective crop cultivation or difficulties with respect to restoration measures.

The differences in type of area (origin and soil properties) as well as origin of the vegetation constitute initial conditions for succession. The assumption was made that these differences will cause different succession trajectories. Thus, distinct phases of succession will occur, which are characterised by differences in the speed of succession for the different area types.

Study sites of different age were selected for each of the area types. On forest soil 14 study sites with an age ranging from 21 to 119 years were established. On post-agricultural soil 13 study sites characterised by natural regeneration of pine with an age ranging from 0 to 64 years and 69 study sites on which pine was planted with an age ranging from 2 to 119 years were studied. On the ash heap 3 study sites with an age ranging from 8 to 14 years and on the mining heap 4 study sites with an age ranging from 3 to 23 years were established.

Beetles were collected from 2004–2006. Thus, for each study site three datasets of consecutive age were produced. In each year sampling was done during the whole vegetation period using pitfall traps (Barber 1931). Since the study sites differed significantly in size, different numbers of pitfall traps were installed. A detailed description of the study sites and the sampling design is provided by Schwerk (2008) (for a detailed description of the study sites see also Appendix I: Description of the study sites).

In the study sites on forest soil and post-agricultural soil, sampling took place from mid May to mid September, and for study sites on the ash and mining heaps, the sampling periods were mid April to mid October (2004) or the end of April to mid October (2005 and 2006), respectively.

### Statistical analysis

Mean individual biomass (MIB) of Carabidae (Szyszko 1990; Szyszko et al. 2000) was applied to assess the stages of succession. MIB is calculated by dividing the biomass of all sampled carabids by the number of specimens caught. According to the observation that in habitats of advanced stages of succession carabid species with large individuals become dominant, MIB increases as succession progresses. An inverse relationship between body size of carabids and degradation of habitats has been demonstrated by several authors (e.g. Blake et al. 1994; Magura et al. 2006). MIB has already been applied successfully in different European countries (e.g. Szyszko et al. 1996; Serrano and Gallego 2004; Schwerk et al. 2006; Cárdenas and Hidalgo 2007).

MIB becomes inaccurate with decreasing numbers of individuals. Therefore, MIB values calculated from samples with less than 25 individuals are indicated in the figures.

Correlations between MIB values and age of the study sites were tested using the Spearman rank correlation coefficient (Sachs 1984).

## Results

### MIB values

**Figure 1.**
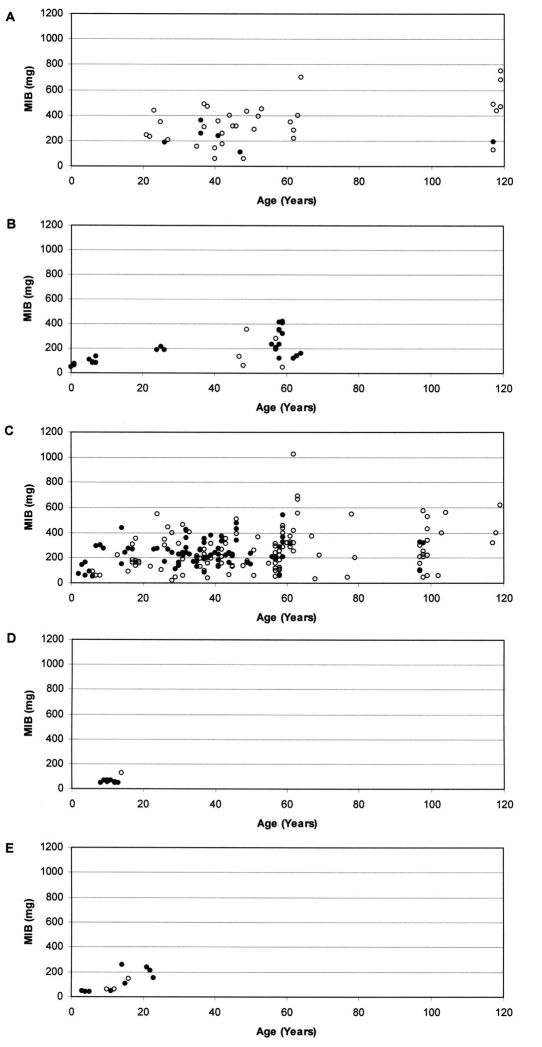
Relationship between MIB values (mg) and age of stands in **A** planted stands on forest soil (*r* = 0.343, *p* <0.05), **B** naturally regenerated stands on post-agricultural soil (*r* = 0.677, *p* <0.001), **C** planted stands on post-agricultural soil (*r* = 0.238, *p* <0.001), **D** stands on ash heap (*r* = 0.025, n.s.) and **E** stands on mining heap (*r* = 0.839, *p* <0.001) (Open circles indicate that MIB was calculated from less than 25 individuals).

Based on a total of 23,602 individuals of carabid beetles, MIB values were calculated for the various study sites. Fig. 1 shows the relationship between the MIB values and age of the study sites for the different types of degraded areas.

At stands on forest soil (Fig. 1A) MIB values range from about 50 mg to about 700 mg. Despite some variability, which may be due to the fact that most values were calculated from less than 25 individuals, values are generally high. Even if young study sites were not included in the study, there is a significant increase of MIB with age of the stands (Spearman rank correlation coefficient, *p* <0.05). At an age of about 20–40 years, MIB values were at about 300 mg, but in very old stands, values were above 400 mg (mean).

The naturally regenerated stands on post-agricultural soil (Fig. 1B) show values from about 50 mg to about 400 mg. The significant increase of MIB values with age of stands (Spearman rank correlation coefficient, *p* <0.001) was comparatively even. At very young age, MIB values were below 100 mg, at an age between 5 and 10 years they were about 100 mg, at an age of about 25 years they were about 200 mg, and at an age of about 60 years MIB values were about 250 mg.

Concerning planted stands on post-agricultural soil (Fig. 1C) MIB values range from about 50 mg to about 600 mg. There is only one exception (MIB >1,000 mg). Here, MIB values increase significantly with age of stands (Spearman rank correlation coefficient, *p* <0.001). Despite high variation within the data a rapid increase during the early phase is visible. At very young age, some MIB values exceed 100 mg and at an age of 10 years some values already exceed 200 mg. However, after about 20 years of forest age MIB values seem to stay at a constant level (a mean of about 250 mg).

On the ash heap (Fig. 1D) almost all MIB values are below 80 mg up to a forest age of almost 15 years, which indicates a delay in succession. The one exception, with a value of about 120 mg, was calculated based on less than 25 individuals. Here we observe no correlation between MIB values and age of stands (Spearman rank correlation coefficient, n.s.).

At the mining heap (Fig. 1E) values range from below 50 mg to about 250 mg. Up to about 15 years of forest age all values fall far below 100 mg, indicating a delay in succession. A significant increase of MIB values with age of stands can be observed (Spearman rank correlation coefficient, *p* <0.001), mainly due to an increase at about 15 years.

### Construction of a conceptual model

Despite remarkable variability for some of the types of study areas, characteristic differences between the area types can be observed. In advanced stages of succession MIB values were highest in planted stands on forest soil and lowest on the brown coal mining heap. Planted stands on post-agricultural soil show a much faster process of succession during the early phase compared to naturally regenerated stands on the same soil type. The post-industrial areas are characterized by an initial delay of succession. These differences suggest that the initial conditions (origin of the soil, origin of the vegetation) indeed seem to be important parameters determining the future trajectory of succession. However, the high variability, particularly in the post-agricultural areas, implies that other parameters are important, too. Especially landscape-related aspects, e.g. distance from resource habitats of colonizing species, may play a role. E.g., Koivula et al. (2002) showed that the degree of heterogeneity of forest landscapes affects the catches of forest carabids.

Based on these results the process of succession may be simplified to the following elements: An ‘initial degradation level’, a possible ‘delay phase’ in the beginning of succession, a more or less ‘steep’ phase of progress of succession (‘increase phase’) and a convergence to a kind of ‘recovery level’.

**Figure 2.**
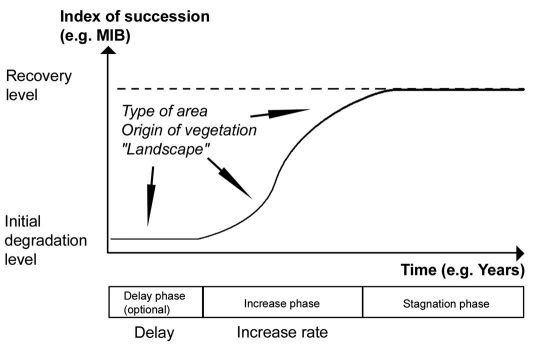
Schematic illustration of the model. Succession starts from an ‘initial degradation level’, followed by a facultative ‘delay phase’ (characterized by the time of ‘delay’). The optional ‘delay phase’ is followed by an ‘increase phase’ (characterized by the ‘increase rate’), and a ‘stagnation phase’ in which the succession process runs towards a ‘recovery level’. The type of area, origin of vegetation, and landscape-related aspects are assumed to influence the trajectory of succession.

Fig. 2 presents a model integrating the different factors and phases. The important factors are type of area (particularly soil characteristics) and origin of vegetation, but also landscape-related aspects (sources for settling species, etc.) may have a reasonable impact (Schwerk 2008). The ‘initial degradation level’ should depend on area type, type of treatment before degradation and type of degradation. At the ‘initial degradation level’ there might be a ‘delay phase’, depending on the type of area to a high degree, but also on the treatment (origin of vegetation) and surrounding landscape. An important characteristic of the ‘delay phase’ is its duration. The subsequent ‘increase phase’ is characterized by its speed, i.e. the steepness of the curve. It should be influenced by similar factors as the ‘delay phase’. Finally, we may define a ‘stagnation phase’ in which the succession process runs towards the ‘recovery level’. The ‘recovery level’ may be identical to the predisturbance level (Majer 1989). As important factors influencing the ‘recovery level’, soil types and surrounding landscape may be assumed.

### Quantification of the model

A quantification of the model can be done using MIB to represent the state of succession. For the construction of a quantitative model it is necessary to comprise mathematically (1) the ‘initial degradation level’, (2) the possible ‘delay’ in succession, (3) the ‘increase rate’ (acceleration), and (4) the deceleration or ‘recovery level’.

The logistic equation (Eq. (1)) has been frequently used to describe growth processes, for example growth of populations (e.g. Begon and Mortimer 1986; for a review on logistic growth models see Tsoularis and Wallace 2002).

*dN*/*dt* = *rN*(*K*-*N*)/*K* (1)

Taking into account parameters 1, 3, and 4 mentioned above, the logistic equation may be useful to describe the process of succession observed in the study sites. *N* may be regarded as the state of succession defined by the ‘initial degradation level’, *r* may be regarded as ‘increase rate’, and *K* as ‘recovery level’. An additional parameter has to be included, however, which describes a possible ‘delay’ at the beginning of the successional process (parameter 2).

To receive exact results the analytical solution of Eq. (1) must be used, which is provided by Wissel (1989) as follows:

*N* = *ertc*/(1+*ertc*/*K*) (2)

with

*c* = *N*(*t*=0)/(1-*N*(*t*=0)/*K*) (3)

To describe the ‘delay’, a ‘starting time’ *t*start = 0 and a ‘delay time’ *t*delay will be defined. Further we define:

*t* = *t*start – *t*delay (4)

At *t* <0 the value of *N* remains at the ‘initial degradation level’, whereas at *t* >= 0 the analytical solution (Eqs. (2) and (3)) is valid.

**Figure 3.**
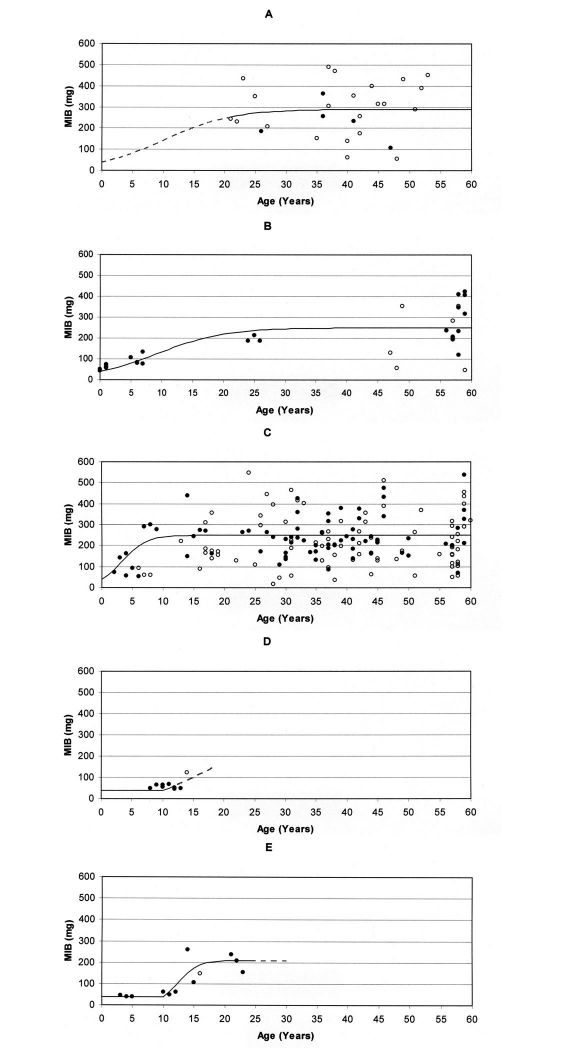
Model graphs based on the parameters in Table 1 for the relationship between MIB values (mg) and age of stands for the first 60 years in **A** planted stands on forest soil, **B** naturally regenerated stands on post-agricultural soil, **C** planted stands on post-agricultural soil, **D** stands on ash heap, and **E** stands on mining heap (Open circles indicate that MIB was calculated from less than 25 individuals; broken lines indicate that the respective part of the graph cannot be verified due to a lack of data).

Applying Eqs. (2), (3) and (4), values for the respective parameters may be specified for the process of succession in the different area types. A graph visualising the run of succession can be easily drawn by selecting values for the four parameters. Fig. 3 shows the respective graphs together with the MIB values for the area types during the first 60 years of forest age, using the values provided in Table 1. These values, which have been chosen in approximation to the empirically elaborated MIB values, seem to result in a suitable description of the respective successions. Table 1 shows clear differences for most of the parameters, particularly the ‘delay’ and ‘increase rate’.

Based on the MIB data an ‘initial degradation level’ of 40 mg (MIB) was chosen for all analysed area types. Due to a lack of data this value cannot be verified for stands on forest soil. For stands on forest soil as well as post-agricultural soil no ‘delay phase’ is given, a fact which is impossible to verify for the forest stands, too. A ‘delay’ exists on both post-industrial areas with a value of about 10 years on the mining heap. The ‘increase rates’ differ strongly. Due a to lack of young study sites it is difficult to specify this value for stands on forest soil. Therefore, the same value as for the naturally regenerated stand on post-agricultural soil was chosen .5. The increase is even faster on the mining heap (value 0.6). Due to a lack of data after 14 years this value cannot be specified for stands on the ash heap (the dashed line in Fig. 3D is based on a theoretical value of 0.28, see Table 1). The highest ‘recovery level’ is observed for stands on forest soil. Taking into account the first 60 years, a ‘recovery level’ of 290 mg (MIB) was chosen. Both naturally regenerated as well as planted stands on post-agricultural soil show a mean ‘recovery level’ of about 250 mg (MIB) after 60 years. The lowest value exists for the mining heap with 210 mg (MIB). Due to a lack of data this value cannot be determined for the ash heap.

**Table 1.** Parameters used for the model graphs in Fig. 3 (Question marks indicate that the respective value cannot be verified due to a lack of data).

*Parameter*	*Planted stands on forest soil*	*Natural regenerated stands on post-agricultural soil*	*Planted stand on post-agricultural soil*	*Stands on ash heap*	*Stands on mining heap*
Initial degr. Level, MIB (mg)	40?	40	40	40	40
Delay, time (Years)	0?	0	0	10?	10
Increase rate, (time-1)	0.18?	0.18	0.5	0.28?	0.6
Recovery level, MIB (mg)	290	250	250	210?	210

## Discussion and conclusion

The successional patterns in the present study are in line with runs of succession described in other publications. Schwerk (2008) compared the trajectory of succession on the ash heap and the mining heap with data obtained in a study on a colliery spoil heap in Germany and pointed out clear analogies. Majer (1989) reported a strikingly similar pattern with respect to data on thrip (Thrysanoptera) recolonisation of Illinois surface-mine spoils. Paquin (2008) identified four groups of species in the succession in black spruce forests of Eastern Canada after forest fire, which characterise different phases of succession. The first three of them seem to fit the successional phases described in the present study.

The mathematical description of the patterns of succession by help of the four parameters revealed similarities as well as differences among the different types of areas.

The same ‘initial level’ after degradation was chosen for the different area types. However, particularly on forest soil the ‘initial level’ might be elevated when compared to the other study sites. For example, Skłodowski (2007) showed less pronounced degradation of forest habitats after a windbreak, probably due to good condition of soil litter. Szyszko (1990) described different degrees of degradation after clear-cuts. Broen (1965) compared clear-cut areas on sandy soil with those on loamy soil. He showed differences in species composition between these two types, with a higher number of forest species in the latter.

A ‘delay’ at the beginning of succession seems to be characteristic for post-industrial areas, particularly those showing primary succession. In the study of Majer (1989) a rapid increase in the number of species took place between 10 to 20 years after the start of succession. Prach et al. (1993) demonstrated a very low initial rate of succession on spoil heaps resulting from brown coal mining. A delayed ecosystem development of the ecosystem on post-industrial areas has been reported also by (Bradshaw (1984, 1987).

According to Horn (1980) succession proceeds rapidly when every species can replace every other species more or less stochastically. Succession proceeds slowly when species of its early stages first have to change environmental conditions for colonization of subsequent species. Since many degraded areas are characterised by poor environmental conditions, the facilitation model described by Connell and Slatyer (1977) seems to be of special importance on these types of areas. A limiting factor seems to be nutrients, particularly a lack of nitrogen (Bradshaw 1984). Depending on previous agricultural practices nutrient contents may vary strongly among post-agricultural areas. This may explain the comparatively high variability in MIB values observed in this type of areas. The higher ‘increase rate’ in planted stands on post-agricultural soil compared to naturally regenerated ones may be explained by a facilitation of succession due to the artificial introduction of pine. However, rates of early forest succession may also differ between different regions as was shown by Yang et al. (2005).

The highest ‘recovery level’ on forest soil indicates most advanced regeneration at these study sites. In accordance with these results, several studies (Szujecki et al. 1983; Szyszko 1990; Skłodowski 1999) have shown that pine stands on forest soil are more developed than stands on post-agricultural land. On the contrary, study sites on the mining heap showed the lowest ‘recovery level’. Schwerk (2008) discovered even lower MIB values on a colliery spoil heap in Germany after about 40 years. Nagler and Wedeck (1998) reported that forest areas on a brown coal mining heap in western Germany were not in a natural forest state after 20–30 years. In addition, Majer (1989) shows that the number of thrip species on surface mine spoils does not reach the level of control areas after 32 years.

The presented model is of practical value because the four parameters facilitate comparisons of successional processes in different areas. A deviation from desired trajectories of succession, e.g. those in undisturbed reference areas, may point to the need for intervention. Based on some years of monitoring MIB values in a given study site, prognoses may become possible, supported by data from comparable areas. For example, the succession on the ash heap will possibly proceed similar to the succession on the colliery spoil heap in Germany (Schwerk 2008). Furthermore, it is possible to transfer the model to other indicators, which provide numerical values to represent the state of succession. Thus, the model can be used in the context of studies that lack data on carabid beetles (either because carabids were not studied or because of habitats where carabids do not occur, e.g. aquatic habitats).

## Appendix 1

Description of the study sites (doi: 10.3897/zookeys.100.1534.app) File format: Microsoft Excel Spreadsheet (xls).

**Explanation note:** The additional file contains a table with the main characteristics of the study sites (enumeration according to Schwerk 2008, data on age relate to the year 2004, age values of “0” and “1” relate to an active management of the respective study sites).

Copyright notice: This dataset is made available under the Open Database License (http://opendatacommons.org/licenses/odbl/1.0/). The Open Database License (ODbL) is a license agreement intended to allow users to freely share, modify, and use this Dataset while maintaining this same freedom for others, provided that the original source and author(s) are credited.
